# Human Centered Development of the Veteran’s Video Connect Match (VVCMatch) Assessment

**DOI:** 10.1093/geroni/igaf122.1885

**Published:** 2025-12-31

**Authors:** Julie Faieta, Timothy Bober, Steven Handler, Dan Ding

**Affiliations:** University of Pittsburgh, Pittsburgh, Pennsylvania, United States; University of Pittsburgh, Pittsburgh, Pennsylvania, United States; Veterans Health Administration, Pittsburgh, Pennsylvania, United States; University of Pittsburgh, Pittsburgh, Pennsylvania, United States

## Abstract

The Veteran’s Healthcare Administration (VHF) was an early adopter of broad telehealth use, however, telehealth implementation within the VHF continues to be limited by digital literacy barriers. Many of the Veterans who would benefit most from the option of telehealth due to heath needs and contextual barriers are also those who carry very limited technology experience. Individualized, and Telehealth-specific digital literacy, evaluation is needed to guide successful telehealth implementation. Therefore, the Veteran’s Video Connect Match (VVCMatch) was developed through task analysis of the telehealth process. The VVCMatch utilizes both objective cognitive evaluation and a novel self-report assessment of the physical, cognitive, and digital literacy-related competencies needed for successful engagement with telehealth. The objective of our study was to gather preliminary Veteran feedback on the content and structure of the VVCMatch (Aim 1) and to gather initial exploratory data on the ability of the VVCMatch to identify Veterans who require education or support prior to adopting telehealth. Our initial findings point to the VVCMatch’s ability to identify cases in which Veterans are not prepared to use telehealth. However, it also flagged cases in which the Veterans were able to complete a call with fair ability. These findings are being used to improve the specificity of the measure in preparation for larger scale psychometric and efficacy testing.

